# *In vitro* activities of lipopeptides against fluconazole-resistant *Candida auris*

**DOI:** 10.1128/spectrum.01786-24

**Published:** 2025-02-27

**Authors:** Simona Fioriti, Francesco Pallotta, Gloria D'Achille, Oscar Cirioni, Oriana Simonetti, Damian Neubauer, Elzbieta Kamysz, Wojciech Kamysz, Lucia Brescini, Sara Caucci, Giuseppina Caggiano, Andrea Giacometti, Gianluca Morroni, Francesco Barchiesi

**Affiliations:** 1 Department of Biomedical Sciences and Public Health, Polytechnic University of Marche 9294 , Ancona, Italy; 2 Infectious Disease Clinic, Azienda Ospedaliero Universitaria delle Marche 18494 , Ancona, Italy; 3 Dermatological Unit, Department of Clinical and Molecular Sciences, Polytechnic University of Marche, Ancona, Italy; 4 Department of Inorganic Chemistry, Faculty of Pharmacy, Medical University of Gdańsk, Gdańsk, Poland; 5 Laboratory of Chemistry of Biological Macromolecules, Department of Molecular Biotechnology, Faculty of Chemistry, University of Gdańsk, Gdańsk, Poland; 6 Interdisciplinary Department of Medicine, Hygiene Section, University of Bari Aldo Moro, Bari, Italy; 7 Infectious Diseases Unit, Azienda Sanitaria Territoriale 1 Pesaro-Urbino, Pesaro, Italy; University of Debrecen, Debrecen, Hungary

**Keywords:** peptides, fluconazole, *Candida auris*, *in vitro *activity

## Abstract

*Candida auris* has increasingly become a global threat due to its wide range of antifungal resistances as well as its ability to sustain outbreaks in clinical settings. Two lipopeptides, C14-NleRR-NH_2_ (Nel) and C14-WRR-NH_2_ (WR), were evaluated against six fluconazole-resistant *C. auris* isolates. Both molecules showed good antimicrobial activity as demonstrated by MIC determination, time-kill, and microscopy experiments. The peptides were able to inhibit fungal growth, while sub-MIC concentrations of the molecules delayed the growth. Moreover, the combinations of the two peptides with fluconazole demonstrated a reciprocal potentiation by checkerboard and time-kill experiments. Our results showed that antimicrobial peptides could be a promising option for the treatment of antifungal-resistant *C. auris*.

**IMPORTANCE:**

As well as antibiotics, also in fungal infections, antimicrobial resistance increased over the years. Moreover, in the last years, a new species emerged, *Candida auris*, as a nosocomial pathogen. *C. auris* possesses intrinsic resistance to common antifungals, such as azoles, that complicate therapeutic options. The combination of these two elements poses a risk for the treatment of fungal infections in the next years. The search for novel compounds with antimicrobial properties is crucial for the treatment of infections to overcome the increasing resistance of these etiological agents.

## OBSERVATION

Infections sustained by fungal pathogens are concerning due to increasing incidence and rates of antifungal resistance: indeed, in 2022, the WHO published a list of priority fungi that need the development of new drugs (1). Although *Candida albicans* still represent the major fungal pathogen involved in infections, in recent years, *Candida auris* emerged as a novel pathogen. First identified in 2009 and now reported almost worldwide, *C. auris* is an environmental opportunistic pathogen that can colonize patients and clinical settings. After colonization, in risk patients, *C. auris* is responsible for candidemia, device infection, soft tissue infection, otitis, osteomyelitis, myocarditis, meningitis, intra-abdominal infection, ocular infection, and urinary tract infection (2, 3), as well as hospital outbreaks (4). Moreover, *C. auris* is frequently resistant to commonly used antifungal agents: about 90% of isolates are resistant to fluconazole, 30% to amphotericin B, and 5% to echinocandins, with some isolates showing pan-resistance to all classes of antifungal agents (5, 6). Given the low rates of resistance expected, according to the CDC, the first line of therapy is represented by echinocandins (7). However, echinocandins may not be a suitable solution depending on the site of infection (e.g., in the urinary tract or central nervous system infections), and additional therapy with amphotericin and flucytosine may be necessary (2). Given the urgency of new drugs, some new antifungals such as manogepix, ibrexafungerp, opelconazole, and rezafungin are currently in late-stage development to treat *C. auris* infections (8). Besides antifungals, another promising approach that could limit the rapid spread of *C. auris* as a hospital pathogen is the use of antimicrobial peptides (AMPs). They are constituted by 18-50 amino acids and have antimicrobial activity by targeting both intracellular and membranous structures (9). Their *in vitro* activity has already been proven against fungi, both yeast and molds (10–12). Considering the urgency of new drugs to counteract *C. auris* infections, we decided to investigate the antifungal activity of two AMPs, C14-NleRR-NH_2_ (Nel) and C14-WRR-NH_2_ (WR), which previously demonstrated efficacy against *Aspergillus fumigatus* (12). We tested the two AMPs against a set of fluconazole-resistant clinical *C. auris* isolates and evaluated the interaction between triazole and AMPs.

**Fig 1:**
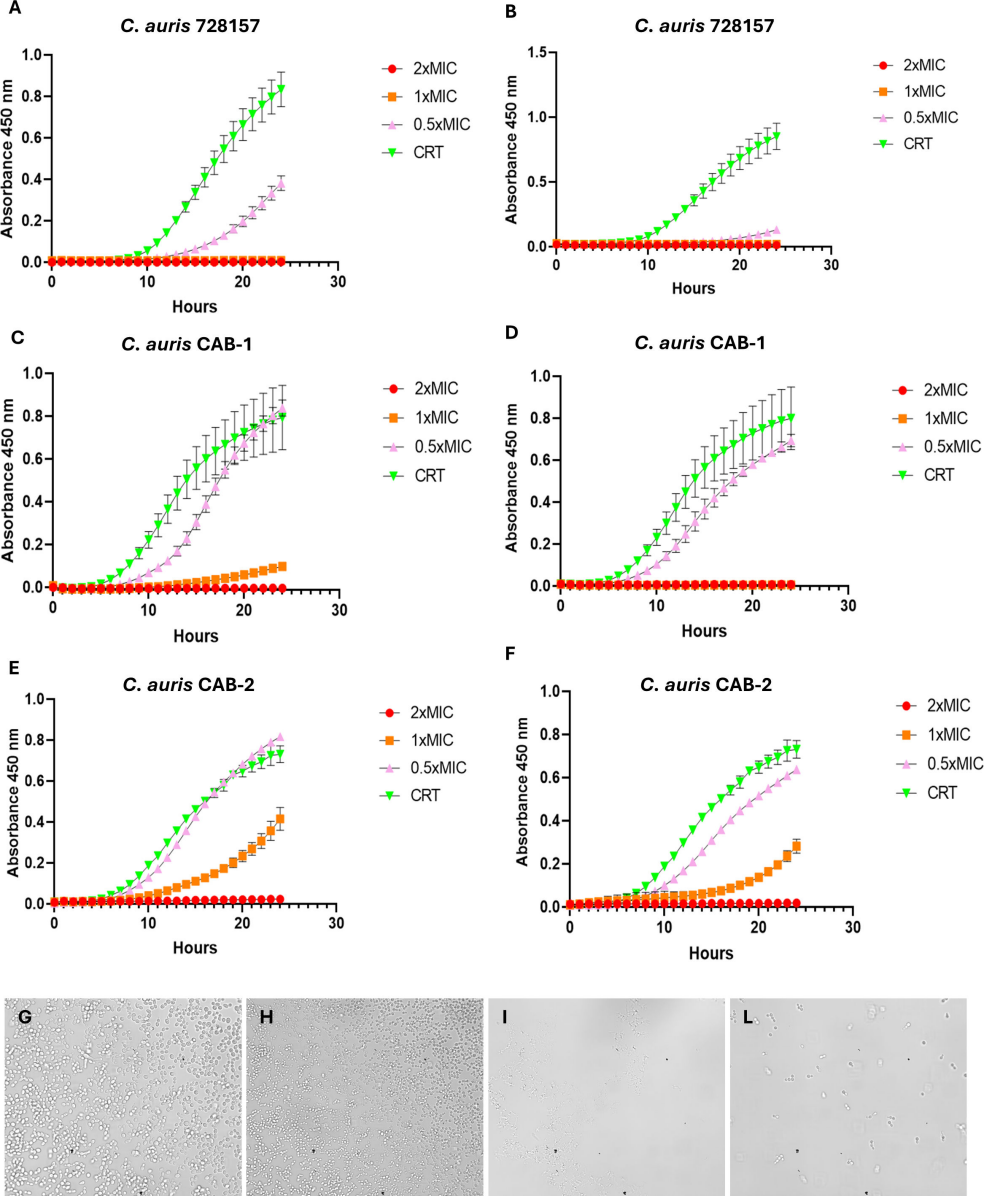
Growth curves and microscopy. (**A**) *C. auris* 728157 treated with NeI (*P* <0.05 after 8 hours of treatment for all the concentrations compared to control); (**B**) *C. auris* 728157 treated with WR (*P* <0.05 after 9 hours of treatment for all the concentrations compared to control); (**C**) *C. auris* CAB-1 treated with NeI (*P* <0.05 after 8 hours of treatment for 1X and 2X MIC concentrations compared to control); (**D**) *C. auris* CAB-1 treated with WR (*P* <0.05 after 9 hours of treatment for all the concentrations compared to control); (**E**) *C. auris* CAB-2 treated with NeI (*P* <0.05 after 7 hours of treatment for 1X and 2X MIC concentrations compared to control); (**F**) *C. auris* CAB-2 treated with WR (*P* <0.05 after 8 hours of treatment for all the concentrations compared to control). CRT, control. Microscopy images with 60× magnification of isolate 728157 after 24 hours of incubation at 37°C: (G) untreated; (**H**) fluconazole 128 µg/mL; (**I**) NeI 0.5X MIC; (**L**) WR 0.5X MIC.

**Fig 2:**
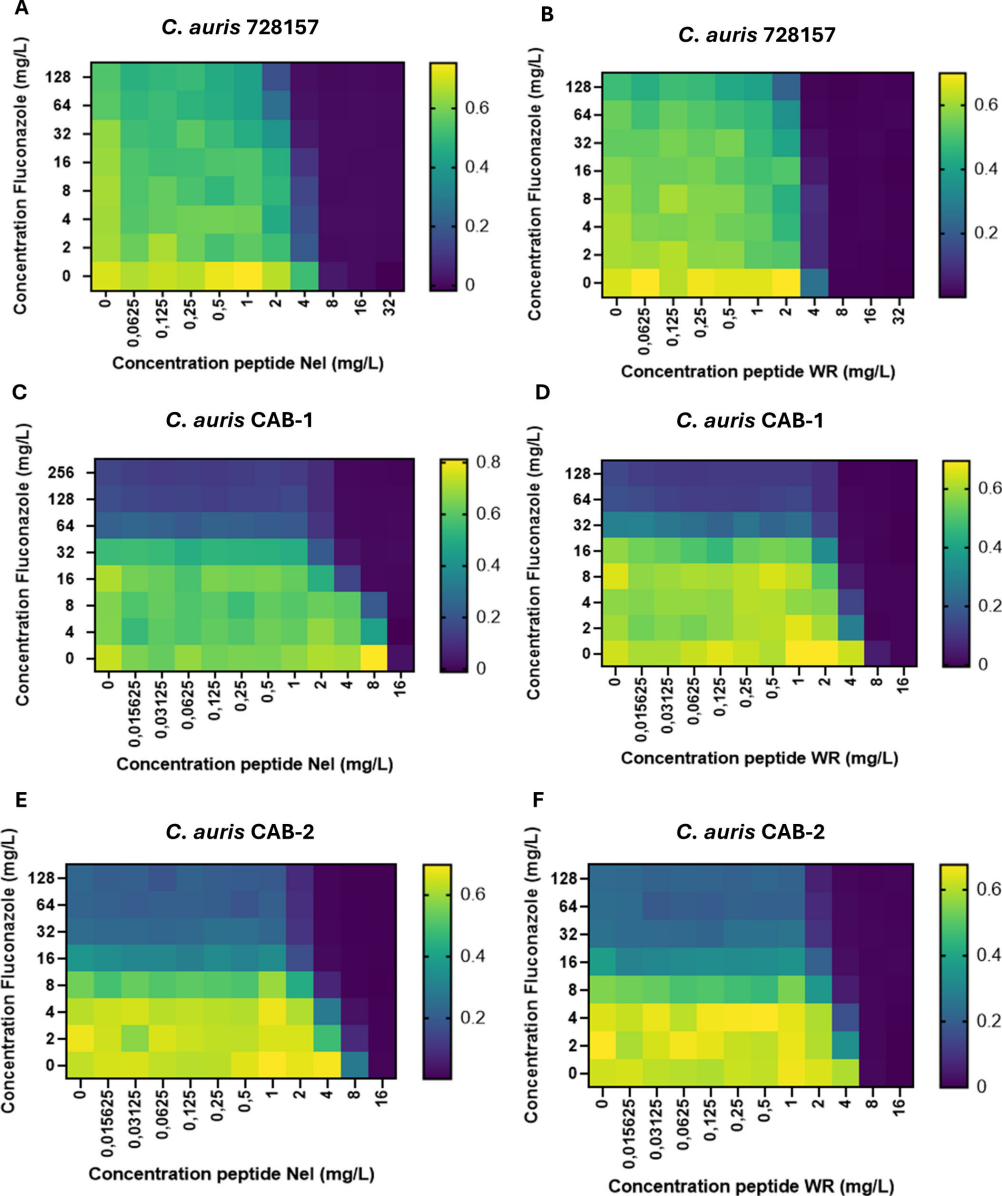
Checkerboard heatmap. (**A**) *C. auris* 728157 fluconazole and Nel combination; (**B**) *C. auris* 728157 fluconazole and WR combination; (**C**) *C. auris* CAB-1 fluconazole and Nel combination; (**D**) *C. auris* CAB-1 fluconazole and WR combination; (**E**) *C. auris* CAB-2 fluconazole and Nel combination; (**F**) *C. auris* CAB-2 fluconazole and WR combination.

Six *C. auris* isolates were collected from clinical specimens and included in the study. Results of MIC determinations, genetic characteristics, and combination experiments are indicated in Table 1. In particular, the six *C. auris* isolates showed MIC values ranging from 32 to >256 mg/L for fluconazole and 8 mg/L for both peptides (9 µM and 8.4 µM for Nel and WR, respectively). NGS analysis showed that single-point mutations in *ERG11* were responsible for the fluconazole resistance: indeed, *C. auris* 728157 harbored the substitution K143R, while the other isolates showed the substitution Y132F; both mutations were already reported in fluconazole-resistant *Candida* isolates (13). Moreover, almost all isolates showed substitutions in TAC1B related to fluconazole resistance (Table 1). All isolates belonged to clade I. To further investigate the activity of the peptides, we selected three *C*. *auris* isolates (728157, CAB-1, CAB-2) to perform additional experiments. Figure 1 shows the growth curves of the three selected isolates exposed to different concentrations of the AMPs. Both peptides completely inhibited *C. auris* 728157 and CAB-1 growth at concentrations of 1X and 2X MIC (Fig. 1A through D), while CAB-2 showed to be less affected by the peptides: in fact, CAB-2 growth was completely inhibited by treatment with both peptides at 2X MIC concentration, but 1X MIC concentration of Nel and WR delayed the start of growth by 14 and 16 hours compared to the control, respectively (Fig. 1E and F). The treatment with 0.5X MIC concentration of NeI and WR delayed the start of growth of *C. auris* 728157 by 16 and 22 hours compared to the control, respectively (Fig. 1A and B), while in the other two strains, these concentrations did not affect the growth (Fig. 1C through F). Treatments with concentrations of 1X and 2X MIC of both AMPs at 24 hours showed a statistically significant difference compared to control. In addition, a 0.5X MIC concentration of WR after 24 hours induced a statistically significant reduction of growth compared to the control. Microscopy analysis confirmed the activity of both AMPs at 24 hours (Fig. 1G, H, I, and L). These delays in the start of microbial growth were already reported for both peptides (12); however, to exclude the possibility that these effects were caused by peptide degradation, we repeated MIC determinations with Nel and WR previously incubated for 24 and 48 hours at 37°C and assessed no changes in MIC values that remain 8 mg/L for both peptides. Then, we decided to test the activity of peptide combinations with fluconazole against *C. auris* strains to increase peptide efficacy and restore susceptibility to fluconazole. Checkerboard assays denoted that NeI exhibited a synergistic activity when combined with the triazole in 3/6 isolates (FICI = 0.500), while WR showed an additive effect in 5/6 yeasts (FICI value from 0.625 to 0.700) and synergistic effect in one strain (FICI: 0.500) (Table 1 and Fig. 2). To validate the results obtained by checkerboard assays, we set up time-kill experiments using the concentration with the best FICI: the time-kill curves showed that treatment with fluconazole in combination with 0.5X and 1X MIC of Nel in *C. auris* 728157 had a synergistic effect, with a log difference of 3.10 and 3.53 at 32 and 48 hours, respectively (Fig. S1A, C, and E). At the same time, the combination of fluconazole and different concentrations of WR had an additive effect (Fig. S1B, D, and E). In *C. auris* CAB-1, fluconazole and subinhibitory concentrations of the two peptides exhibited a log difference of 2.19 and 2.96 compared to single administration when fluconazole was combined with 0.5X MIC of NeI and WR, respectively, after 12 hours (Fig. S2A through C). A lower efficacy of the combination was detected in the CAB-2 isolate, confirming the additive effect resulted from the checkerboard assay; the combination of fluconazole and 0.5X MIC of Nel and WR showed a log difference of 1.19 and 1.69, respectively, after 12 hours (Fig. S2D through F). Regarding the toxicity of the two compounds, a statistically significant reduction of Vero E6 viability compared to control was obtained only after 48 hours of treatment with 32 mg/L of both peptides (Fig. S3).

**TABLE 1:** MIC values, genetic characteristics, and FICI of *C. auris* strains^*a*^

Isolates	MIC (mg/L)					Clade	ERG11 mutations	TAC1 mutations	FICI	
	AMB	ANID	FLU	NeI	WR				FLUxNeI	FLUxWR
728157	0.5	0.25	>128	8	8	I	K143R	R215K, A640V	0.500	0.625
CAB-1	1	0.5	64	8	8	I	Y132F	R215K, A583S	0.500	0.625
CAB-2	1	0.25	32	8	8	I	Y132F	R215K	0.625	0.625
CAB-3	1	0.25	32	8	8	I	Y132F	R215K, A583S	0.625	0.625
CAB-4	1	0.25	32	8	8	I	Y132F	R215K	0.625	0.750
CAB-5	1	0.25	64	8	8	I	Y132F	R215K, A583S	0.500	0.500

^*a*^ AMB, amphotericin; ANID, anidulafungin; FLU, fluconazole; FICI, fractional inhibitory concentration index.

*C. auris* concern is increasing worldwide: this species shows an unfavorable susceptibility profile to common antifungal agents, making it difficult to administer effective treatment. In this context, AMPs can be promising agents in antifungal therapy. Our study expands the knowledge of AMPs as antifungal drugs and confirms that these molecules are effective *in vitro* also against *C. auris*, as was previously reported for other compounds (14–17). We tested two peptides: both showed good antifungal activity against azole-resistant *C. auris*. Similar to echinocandins, AMPs used in our study belong to the class of lipopeptides and have previously shown antifungal activity against *Aspergillus* (12). Furthermore, a reciprocal potentiation, ranging from indifference to synergy, was found when the AMPs were combined with fluconazole. Interestingly, an antagonistic effect was never found. However, our study presents some limitations: currently, the action mechanism of AMPs is unknown, and further studies are needed to identify the molecular mechanisms of their antifungal activity. Furthermore, although promising, our results are preliminary, and no *in vivo* experiments have been done.

Given the concerning spread of *C. auris*, new molecules with antifungal activity are urgently needed. Our results showed that AMPs may represent a possible option to treat *C. auris* isolates, although other studies are warranted to investigate their antifungal activity and their safety in *in vivo* models.

## SUPPLEMENTAL MATERIAL

Fig. S1 spectrum.01786-24-s0001.tiff Time-kill curves and CFUs counts of *C. auris* 728157 with fluconazole (128 mg/L) in combination with peptides.

Fig. S2 spectrum.01786-24-s0002.tif Time-kill curves and CFUs counts of *C. auris* CAB-1 and CAB-2 with fluconazole in combination with peptides.

Fig. S3 spectrum.01786-24-s0003.tif Cytotoxicity of the two peptides on Vero E6 cells.

Supplemental material spectrum.01786-24-s0004.docx Supplemental Materials and Methods; Supplemental figure captions.

ASM does not own the copyrights to Supplemental Material that may be linked to, or accessed through, an article. The authors have granted ASM a non-exclusive, world-wide license to publish the Supplemental Material files. Please contact the corresponding author directly for reuse.
