# Supplementary figures and images for: In vitro activities of lipopeptides against fluconazole-resistant Candida auris
Source: Microbiol Spectr. 2025 Feb 27;13(4):e01786-24. doi: 10.1128/spectrum.01786-24 (PMC11960442; doi:10.1128/spectrum.01786-24)

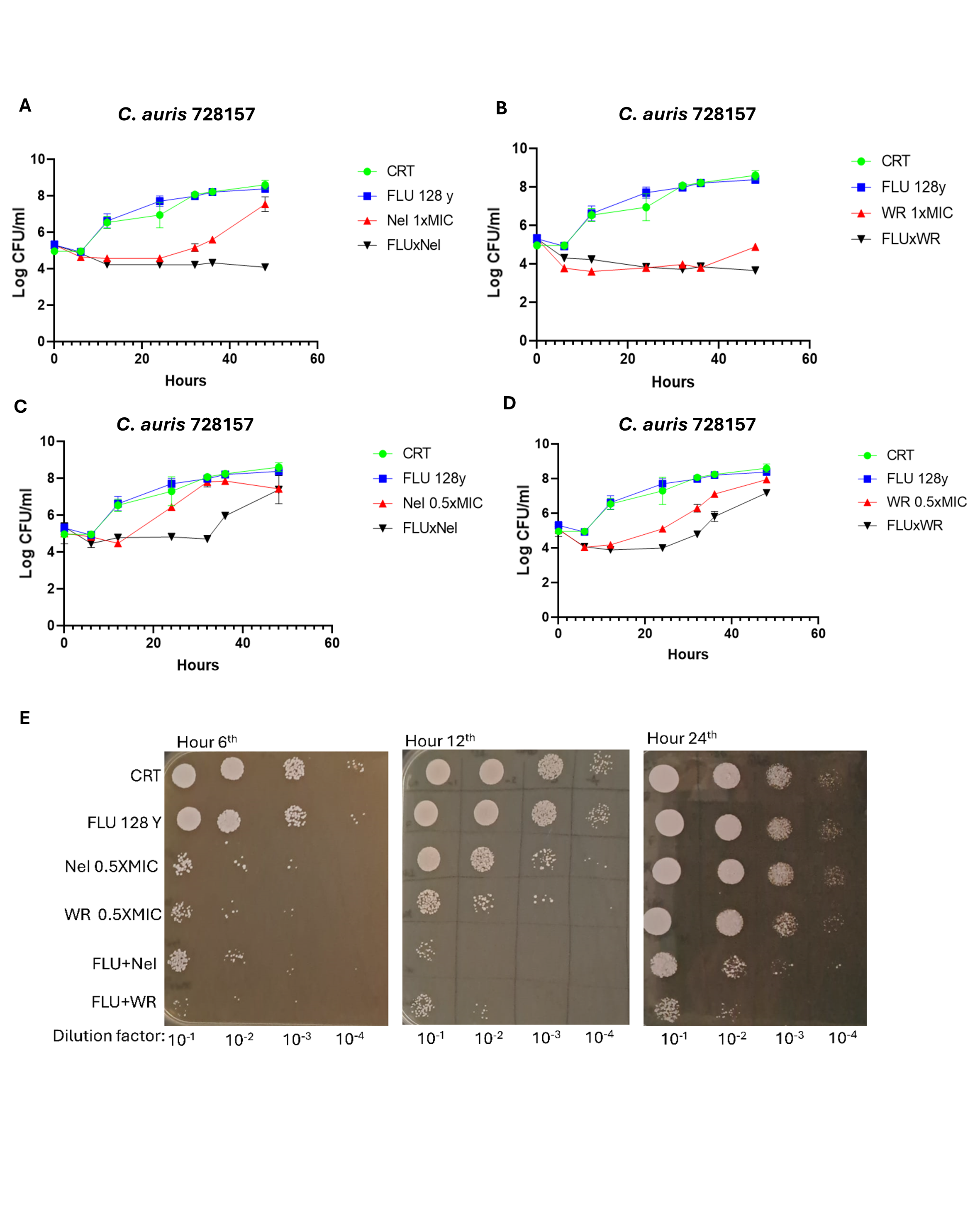

Supplement: Fig. S1 — Time-kill curves and CFUs counts of C. auris 728157 with fluconazole (128 mg/L) in combination with peptides. [file spectrum.01786-24-s0001.tiff]

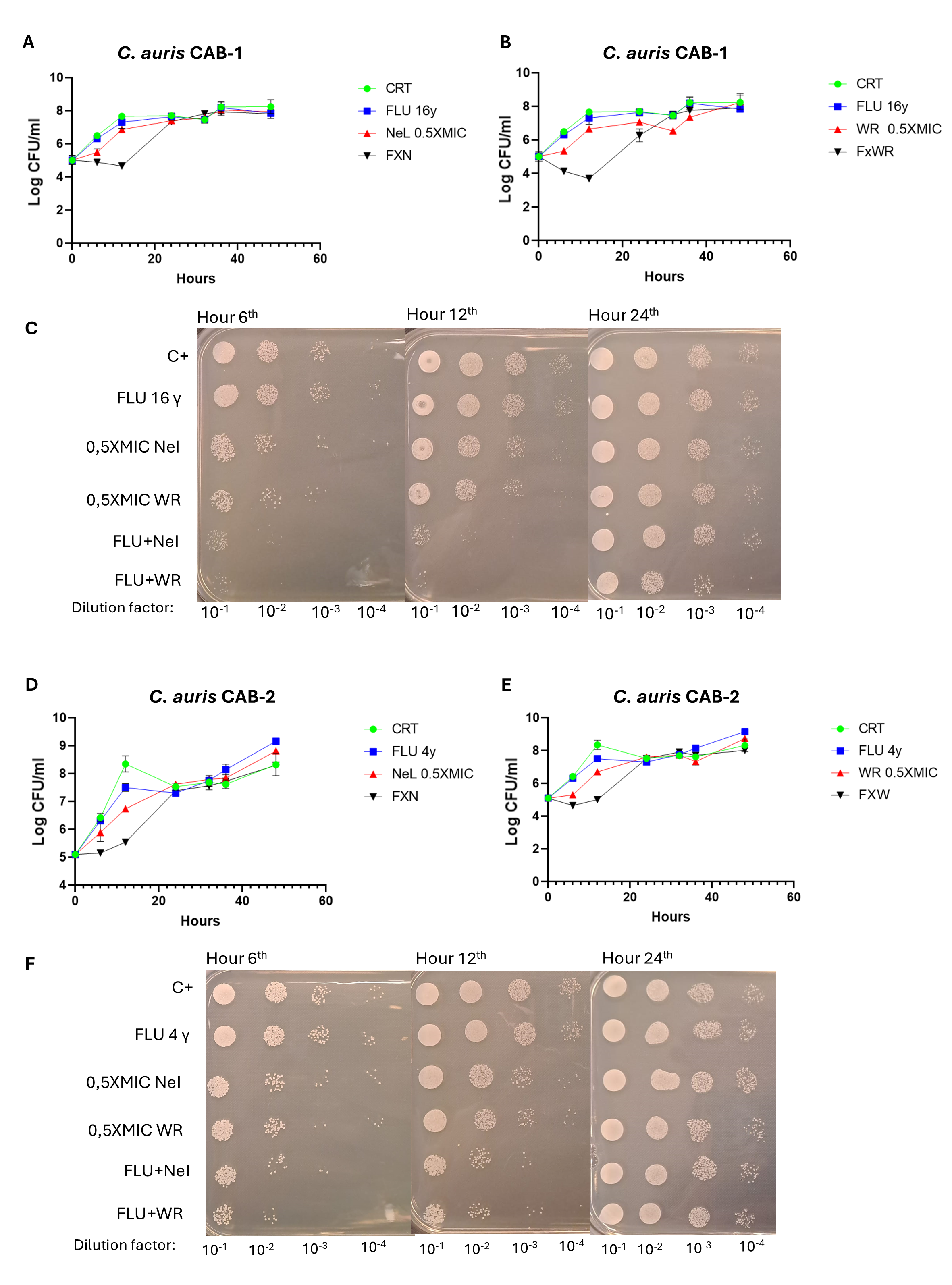

Supplement: Fig. S2 — Time-kill curves and CFUs counts of C. auris CAB-1 and CAB-2 with fluconazole in combination with peptides. [file spectrum.01786-24-s0002.tif]

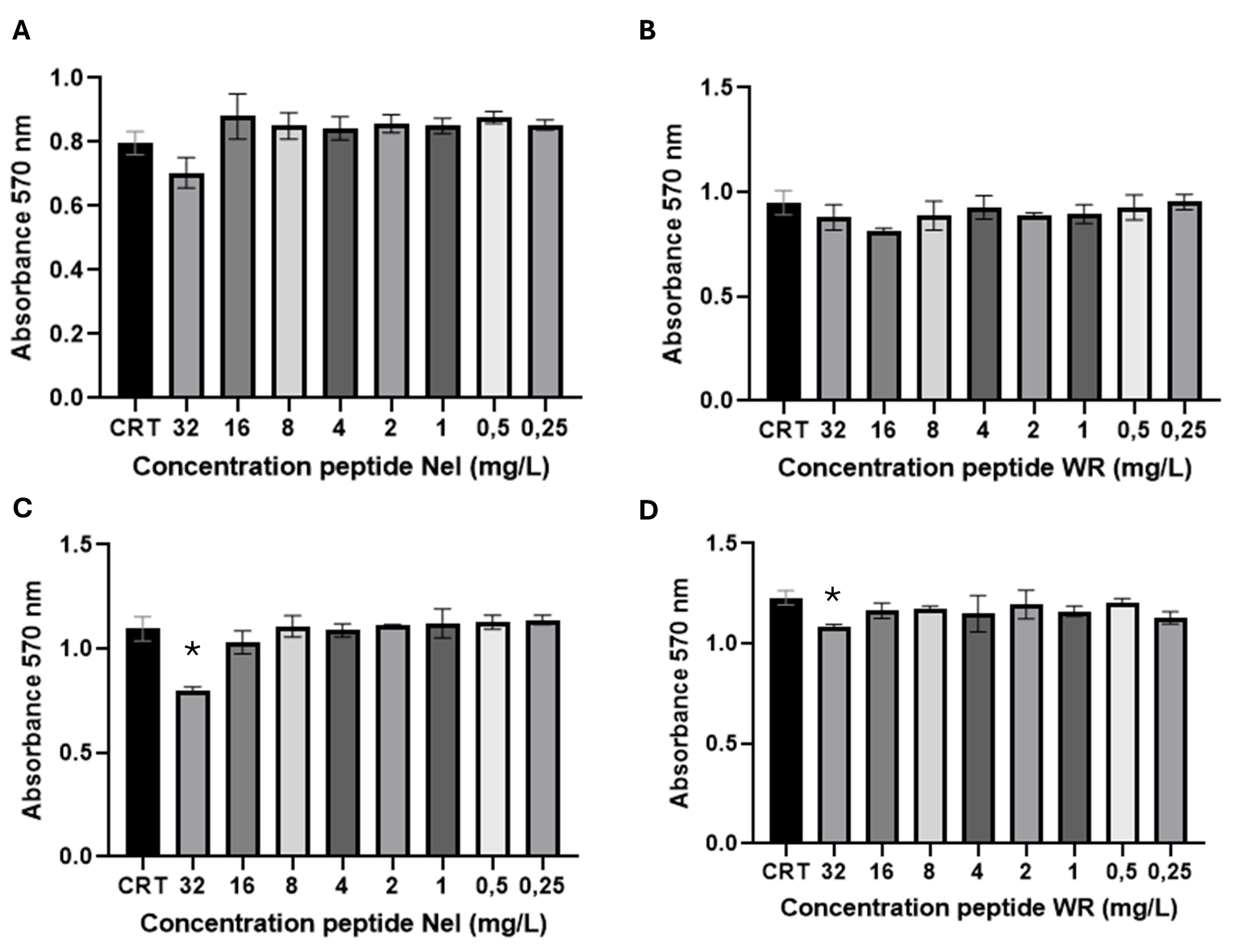

Supplement: Fig. S3 — Cytotoxicity of the two peptides on Vero E6 cells. [file spectrum.01786-24-s0003.tif]
