# Supplementary material for: In vitro activities of lipopeptides against fluconazole-resistant Candida auris
Source: Microbiol Spectr. 2025 Feb 27;13(4):e01786-24. doi: 10.1128/spectrum.01786-24 (PMC11960442; doi:10.1128/spectrum.01786-24)
Supplement: Supplemental material — s and Methods; Supplemental figure captions. [file spectrum.01786-24-s0004.docx]

**Materials and Methods**

*Isolates and WGS.* 5 clinical isolates of fluconazole-resistant *C. auris* were included in the study. The isolates were recovered from different clinical specimens, identified by MALDI-Tof analysis and sequenced on an Illumina Miseq platform (2x250bp). Assembly was obtained using SPAdes v3.15.4 (<https://github.com/ablab/spades>). Analysis of mutations was performed using *C. auris* B8441 as reference genome (acc. No PEKT00000000.2)

*Fluconazole and peptides* Fluconazole was diluted in DMSO. C14-NleRR-NH_2_ (Nel) and C14-WRR-NH_2_ (WR) were synthesized as previously described [1] and diluted in water.

*Determination of MICs.* To determine the minimal inhibitory concentration (MIC) of Fluconazole, Nel, and WR a broth microdilution method was performed following the EUCAST methodology [2]. The media used for this assay was RPMI 1640 with 2% of glucose and the concentration of antifungal and peptides tested ranged from 128 to 0.25 mg/l.

*Checkerboars Assay* The combination between peptides and fluconazole was assessed with the checkerboard method (following the EUCAST recommendations for media, inoculum, antifungals dilutions, and results reading), by interpreting the interactions obtained with the classification suggested by Odds [3]. All experiments were replicated at least three times.

*Growth Curves.* The action of the peptides was assessed by growth curve measuring OD_450_ nm every hour for 24 h using peptides at different concentrations (0.5X, 1X, 2X MIC). The combination of peptides (0.5X and 1X MIC) and fluconazole (128, 16, 4 mg/L concentration) was assessed by growth inhibition assay and counting colony forming units (CFU/ml) in Sabouraud plates at different time points (0, 6, 12, 24, 32, 38 and 48 h). For both types of experiments, 2-5 x 10^5^ CFU/ml were diluted in RPMI-1640 and incubated at 35°C with AMPs, fluconazole, and their combination. Untreated isolates were used as control. All experiments were replicated at least three times. The results were interpreted as suggested by White et al. [4].

*Cell viability assay.* The effect of AMPs on Vero E6 cells (ATCC n° CRL-1586) viability was assessed by a 3-(4,5-dimethylthiazol-2-yl)-2,5-diphenyltetrazolium bromide (MTT) assay (Sigma, St. Louis, MO, USA). Vero E6 cells were seeded in 96-well plates at a density of 26000 cells/well and treated with increasing compound concentration (0.13 - 32 µg/ml). Time-course analyses of cell survival were determined at 24 and 48 hours. Following treatments, the medium was replaced with fresh medium supplemented with MTT at a final concentration of 0.5 mg/ml. After incubation for 2 h at 37 °C, 0.2 ml of dimethyl sulfoxide was added to dissolve formazan crystals formed by live cells and plates were shaken for 10 minutes. Absorbance was measured at 570 nm using a microplate reader. The optical density of untreated cells was considered as 100% viability. The relative cell viability (%) was calculated as (OD_570_ of treated samples/OD_570_ of untreated samples) × 100. Each experiment was performed three times in triplicate.

*Statistical analysis.* Statistical analysis was performed using GraphPad Prism 8 software and ANOVA test. p-values <0.05 were considered statistically significant.

*Mycroscopy* Microscopy analysis was performed with NED-VD microscope (NTP Nano Tech Projects). Pictures were captured after 24 hr of treatment with fluconazole, peptides and combinations of both.

*Stability of compounds* The peptides were incubated at 37°C for 24 and 48 hours. They were then used to perform MIC determination as previously described.

References:

1. Fioriti S, Cirioni O, Simonetti O, Franca L, Candelaresi B, Pallotta F, Neubauer D, Kamysz E, Kamysz W, Canovari B, Brescini L, Morroni G, Barchiesi F. In Vitro Activity of Novel Lipopeptides against Triazole-Resistant Aspergillus fumigatus. J Fungi (Basel). 2022 Aug 18;8(8):872. doi: 10.3390/jof8080872. PMID: 36012859; PMCID: PMC9409728.
2. <https://www.eucast.org/fileadmin/src/media/PDFs/EUCAST_files/AFST/Files/EUCAST_E.Def_7.4_Yeast_definitive_revised_2023.pdf>
3. Odds FC. Synergy, antagonism, and what the chequerboard puts between them. J Antimicrob Chemother. 2003 Jul;52(1):1. doi: 10.1093/jac/dkg301. Epub 2003 Jun 12. PMID: 12805255.
4. White RL, Burgess DS, Manduru M, Bosso JA. Comparison of three different in vitro methods of detecting synergy: time-kill, checkerboard, and E test. *Antimicrob Agents Chemother*. 1996;40(8):1914-1918. doi:10.1128/AAC.40.8.1914

**Supplemental Figure Captions**

**Figure S1.** Time-kill curves of *C. auris* 728157 with fluconazole (128 mg/L) in combination with peptides. A) fluconazole and 1X MIC NeI; B) fluconazole and 1X MIC WR; C) fluconazole and 0.5X MIC NeI; D) fluconazole and 0.5X MIC WR; E) Counting colony forming unit (CFU) at different hour. CRT, control; FLU, fluconazole; y, mg/L.

**Figure S2**. Time-kill curves of *C. auris* CAB-1 and CAB-2 with fluconazole in combination with peptides. A) CAB-1, fluconazole and 0.5X MIC NeI; B) CAB-1, fluconazole and 0.5X MIC WR; C) Counting colony forming unit (CFU) at different hour; D) CAB-2, fluconazole and 0.5X MIC NeI; E) CAB-2, fluconazole and 0.5X MIC WR; F) Counting colony forming unit (CFU) at different hour. CRT, control; FLU, fluconazole; y, mg/L.

**Figure S3.** Cytotoxicity of the two peptides on Vero E6 cells. A) exposition to Nel for 24 hours; B) exposition to WR for 24 hours; C) exposition to Nel for 48 hours; D) exposition to WR for 48 hours. CRT, control; ***** *p*-value < 0.05. The error bars represent the standard deviation (SD)
